# The value of bone marrow, liver, and spleen imaging in diagnosis, prognostication, and follow-up monitoring of myeloproliferative neoplasms: a systematic review

**DOI:** 10.1186/s40644-021-00405-7

**Published:** 2021-04-20

**Authors:** Stefanie Slot, Niels W. C. J. van de Donk, René H. J. Otten, Bouke J. H. Boden, Josée Zijlstra, Pieter G. H. M. Raijmakers, Sonja Zweegman

**Affiliations:** 1 Department of Hematology, Cancer Center Amsterdam, Amsterdam UMC, Vrije Universiteit Amsterdam, De Boelelaan 1117, 1081HV Amsterdam, The Netherlands; 2 Medical Library, Vrije Universiteit Amsterdam, De Boelelaan 1117, 1081HV Amsterdam, The Netherlands; 3 Department of Radiology, Onze Lieve Vrouwe Gasthuis, Oosterpark 9, 1091AC Amsterdam, The Netherlands; 4 Department of Radiology and Nuclear Medicine, Amsterdam UMC, Vrije Universiteit Amsterdam, De Boelelaan 1117, 1081HV Amsterdam, The Netherlands

**Keywords:** Myeloproliferative neoplasms, Myelofibrosis, Diagnostic accuracy, Magnetic resonance imaging, PET/CT, Ultrasound, Computed tomography, Radiography, Dual-energy X-ray absorptiometry

## Abstract

**Background:**

Diagnostic and treatment response criteria for the JAK2/CALR/MPL mutation-related myeloproliferative neoplasms (MPNs) are largely based on bone marrow (BM) biopsy results. However, these biopsies have several limitations, such as the risk of sampling error. Also, the prognostic impact of BM abnormalities is largely unclear. Although not currently used in clinical practice, imaging techniques might offer additional information. In this review, we investigated the value of BM, liver, and spleen imaging for diagnosis, prognostication, and response monitoring of the JAK2/CALR/MPL mutation-related MPNs (i.e. essential thrombocythemia (ET), polycythemia vera (PV), and myelofibrosis (MF)).

**Methods:**

A systematic literature search was performed via PubMed, Embase and the Cochrane Library up to 2020 March 26th. Of 5505 identified records, 55 publications met the eligibility criteria (i.e. containing original data on the imaging appearance of BM, spleen, or liver in adult ET, PV, or MF patients, published in a peer-reviewed journal, written in English).

**Results:**

Many explorative studies described imaging features, sometimes with comparisons to clinical characteristics. Studies reporting measures of diagnostic accuracy included 1) splenic transient elastography to predict BM fibrosis grade in MF, 2) dynamic contrast-enhanced MRI to discern MF patients from ET patients and healthy controls, and 3) 18-fluorodeoxyglucose PET to detect residual disease after stem cell transplantation in MF. The diagnostic accuracies of radiography and ^99m^Tc-colloid scintigraphy were derived from several other articles. Except for the study on 18-fluorodeoxyglucose PET, we established substantial concerns regarding risk of bias and applicability across these studies, using the QUADAS-2 tool. Three publications described a correlation between imaging results and prognosis, of which one quantified the effect.

**Conclusions:**

Based on current data, MRI (T1-weighted/STIR, Dixon) seems especially promising for the evaluation of BM fat content - and indirectly cellularity/fibrosis - in MF, and possibly for estimating BM cellularity in ET/PV. 18-fluorodeoxyglucose and 18-fluorothymidine PET/CT might be useful for evaluating BM fibrosis, with good reported accuracy of the former for the diagnosis of residual disease. Further research on these and other techniques is warranted to determine their exact value. Future researchers should improve methodology and focus on evaluation of diagnostic accuracy and prognostic implications of results.

**Supplementary Information:**

The online version contains supplementary material available at 10.1186/s40644-021-00405-7.

## Background

Essential thrombocythemia (ET), polycythemia vera (PV), and myelofibrosis (MF) are relatively rare diseases that belong to the group of myeloproliferative neoplasms (MPNs). These diseases - with age-standardized incidence rates of 1.6, 1.48, and 0.52/100,000 person-years, respectively - greatly affect hematopoiesis [1]. Normally, hematopoiesis occurs in the liver and spleen in the fetus, occupies most of the bone marrow (BM) at birth, and is confined to the axial skeleton in adulthood [2]. Conversion from cellular to fatty marrow during childhood starts in the distal extremities and proceeds proximally. In adults, fatty marrow contains 80% fat, compared with 40–60% in cellular marrow. High hematopoietic demand can cause reconversion to cellular marrow. The marrow framework is formed by cancellous bone, composed of primary and secondary trabeculae. Blood supply comes from periosteal and nutrient arteries, the latter forming a sinusoidal network [3]. In MPNs, various alterations in BM composition develop over time, presumably caused by driving mutations and inflammatory cytokines. ET, PV, and MF share similar driving mutations and are therefore grouped together as the JAK2/CALR/MPL mutation-related MPNs [4]. Although these MPN subtypes form distinct disease entities, they share morphologic features and have the ability to transform into each other. Classically, ET and PV are characterized by megakaryocytic- and trilinear hypercellularity, respectively. Myeloid hypercellularity can occur in early MF, progressing to atrophy with fibrosis and osteosclerosis in later stages [2]. Neoangiogenesis is found in all three diseases, albeit most pronounced in MF [5, 6]. BM alterations can coincide with constitutional symptoms and hepatosplenomegaly, due to a shift of hematopoiesis to the liver and spleen. Apart from spleen size measurements, imaging is currently not routinely used in the management of ET, PV, and MF. However, several challenges regarding diagnosis, prognostication, and response monitoring exist, for which imaging might form a solution.

Diagnostic criteria in ET, PV, and MF are largely based on BM biopsy results [4, 7]. However, overlapping histopathological features are known to complicate discrimination of these MPN subtypes. Also, BM biopsies yield limited information on functional processes - such as osteoblast activity and BM blood flow -, and offer no information on other hematopoietic compartments including the liver and spleen. Lastly, the reliability of single BM samples is questionable given the occurrence of crush artefacts [4], ‘sampling error’ in case of non-homogeneous disease distribution [8], and large interobserver variability regarding fibrosis grading [4]. Imaging might aid in diagnosis of MPN subtypes and abnormalities in the hematopoietic compartment, through increased spatial resolution and/or visualization of dynamic processes.

Apart from allogeneic stem cell transplantation (allo-SCT) – which is associated with high treatment-related morbidity and mortality -, no curative treatments exist for the JAK2/CALR/MPL mutation-related MPNs. Current treatment response criteria mainly assess clinical and laboratory parameters, but a complete response also requires a total reversal of BM abnormalities [7, 9]. However, significant BM changes are not often found on the short term and the invasive nature of the BM biopsy limits serial monitoring. Given the ongoing development of novel – often expensive - drugs, alternative techniques for (early) assessment of changes to the hematopoietic compartment during treatment are desirable.

Lastly, prognosis in the JAK2/CALR/MPL mutation-related MPNs varies greatly. Whilst most ET patients have a normal life expectancy, median overall survival in MF is approximately six years. Prognostic scoring models in ET and PV are largely based on clinical parameters, whereas mutational analyses play a larger role in MF [10]. Interestingly, the prognostic impact of the various BM abnormalities is largely unclear [9, 11–14], even though normalization hereof is pursued during treatment. Imaging might offer additional insight into prognostic relevance of BM abnormalities, since it can provide information on both focal disease severity and distribution throughout the hematopoietic compartment.

Hitherto, several reviews have described common imaging findings of BM and disease-related complications in MF [15, 16]. However, to our knowledge the value of imaging for diagnosis, prognostication and follow-up monitoring in the JAK2/CALR/MPL mutation-related MPNs has not been systematically evaluated. This was the purpose of our systematic review.

## Methods

We followed the Preferred Reporting Items for Systematic Reviews and Meta-Analysis (PRISMA) statement [17]. Pre-specified and documented inclusion criteria were 1) publications describing the imaging appearance of BM, liver and/or spleen in human ET, PV, or MF patients aged ≥18 years, and 2) original data.. Publications were excluded if they only contained (spleen) size measurements and/or descriptions of MPN-related complications (e.g. focal extramedullary hematopoiesis, thrombosis). For the final review, only peer-reviewed published articles written in English were included and case reports were omitted. No restrictions on publication date or population size were imposed.

SS and RO systematically searched the databases PubMed, EMBASE.com and The Cochrane Library (via Wiley) from inception to 2020 March 26th, using search terms expressing an equivalent of ‘myeloproliferative neoplasm’ and terms comprising different imaging techniques. The full search strategies are listed in the Supplemental Material. Reference lists of eligible studies were searched for additional publications. Results were collected in EndNote. Records were screened by SS to identify potentially relevant publications. Review was then independently performed by two authors (SS plus either ND or SZ) to identify studies that met the eligibility criteria, with disagreements resolved by a third reviewer (SZ or ND). Extracted data from eligible studies were entered into an Excel spreadsheet, including: study aims and design, inclusion criteria, patient number, MPN subtype, modality and timing of imaging, major imaging findings and correlations with histopathology or prognosis. We checked for duplicate publications by juxtaposing author names. All reports on a study were considered and data were pieced together if possible. Data from the report with the largest population were included in case of inconsistent patient numbers. Other relevant inconsistencies are mentioned in the text. Outcome measures of primary interest were diagnostic accuracy (e.g. sensitivity, specificity, positive and negative predictive values, area under the receiver operating characteristics (AUROC) curve), reliability (e.g. Cohen’s *k*) and prognosis (e.g. median survival).

SS and SZ assessed diagnostic accuracy studies for risk of bias and applicability, using the QUADAS-2 tool [18]. The review question was: what is the accuracy of imaging techniques that visualize (components of) the BM, spleen and/or liver in ET, PV, or MF patients (according to conventional criteria, i.e. WHO or equivalent), regarding determination of disease type and/or characteristics (compared to diagnostic criteria and/or histopathological examination) or monitoring of therapy response (compared to conventional response criteria)?

## Results

### Study selection

**Fig. 1:**
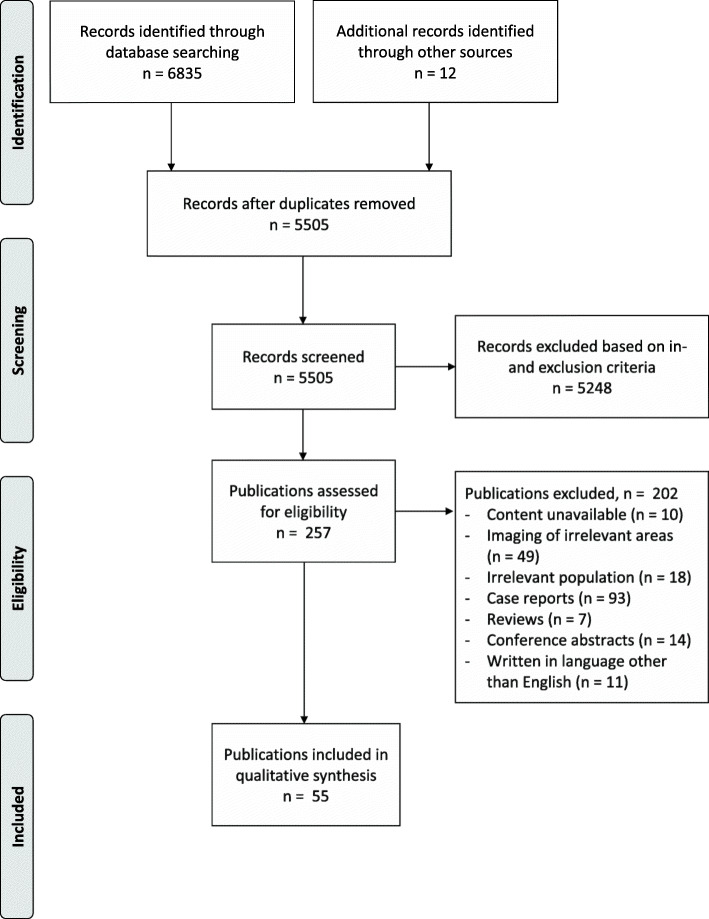
Flow of studies through selection process

Figure 1 depicts details of the selection process.

### Study characteristics

Out of 5505 records, 55 publications were eligible for inclusion. Many different techniques were used, including forms of ultrasonography (four studies, *n =* 176 MF) and quantitative computed tomography (qCT) (three studies, *n* = 24 MF/31 PV/14 ET), dual-energy X-ray absorptiometry (DXA) (four studies, *n* = 33MF/31 PV/14 ET), radiography (seven studies, *n* = 223 MF/41 PV), magnetic resonance imaging (MRI) (15 studies, *n* = 115 MF/33 PV/27 ET), scintigraphy (17 studies, *n* = 273MF/158PV), single photon emission CT (SPECT) (one study, *n* = 6 MF), positron-emission tomography (PET) (six studies, *n* = 60MF/6PV) and thermography (one study, *n* = 6 MF).

Below we present a general summary of imaging findings, followed by a synthesis of evidence regarding the use of imaging in diagnosis, prognostication, and follow-up monitoring. Concluding remarks concerning separate imaging modalities are listed in Table 1. Study details can be found in the Supplemental material.

**Table 1:** Summary of findings

Modality	Main findings
**Ultrasound**	- Normal echotexture does not rule out splenic or hepatic myeloid metaplasia in MF (da-) - Focal splenic myeloid metaplasia and solid tumor metastases can show a similar appearance (da-)
**Elastography**	- Increased hepatic stiffness in MF possibly reflects myeloid metaplasia (da-) - Splenic elastography might aid in differentiating MF patients from healthy controls (da-) ^b^ Splenic stiffness combined with clinical parameters might be able to identify MF patients with grades 0–1 or grades 2–3 fibrosis **(accuracy of 97% and 94–95% at best cut-off values; Iurlo et al.)** - Changes in splenic stiffness have been observed during cytoreductive treatment
**Splenic sound speed measurement**	- Was not found to differentiate all MF patients from healthy controls or patients with other causes of splenomegaly (da-) - Splenic sound speed might indirectly reflect BM fibrosis grade in MF, as well as changes herein during treatment (da-)
**Splenic attenuation**	- Was not found to differentiate MF patients from healthy controls (da-)
**DXA**	- Was not found to differentiate ET and PV patients from healthy controls (da-) - An increased Z-score probably reflects osteosclerosis in MF (da-)
**Radiography**	^**b**^ **Is** probably insufficient in detecting or excluding the presence of osteosclerosis in MF **(derived sensitivities 60–100%, derived specificities 43–61%; Pettigrew/Hickling/Pawelski et al.)**^b^ - The presence of sclerosis probably rules out pure hypercellular disease (da-)
**CT**	- HR-pQCT was not found to differentiate ET and PV patients from healthy controls (da-) - HR-pQCT might be able to identify MF patients with advanced osteosclerosis (da-) - qCT might reflect the degree of osteosclerosis in MF (da-)
**MRI -** **T1-weighted imaging and STIR**	- The skeletal T1 signal reflects BM fat content, and thereby probably indirectly reflects BM cellularity in ET/PV and cellularity and/or fibrosis in MF (da-) - Skeletal T1 and STIR signals might reflect the presence of fibrosis in MF and changes herein during treatment (da-), but the correlation with histopathology was not unequivocal - T1-weighted imaging of the axial and femoral BM might aid in differentiating MF patients from both healthy controls and ET patients (da-), but a decreased signal does not exclude ET/PV - T1 + STIR signal distribution in the axial and femoral BM possibly corresponds to disease severity and prognosis in MF
**MRI - CSI**	- Skeletal CSI can theoretically indirectly quantify BM cellularity in PV and cellularity and/or fibrosis in MF (da-) and was shown to reflect changes herein during treatment (da-) - Skeletal CSI might aid in differentiating PV and MF patients from healthy controls (da-)
**(D)CE-MRI**	- The CE T1 signal possibly reflects BM perfusion in MF (da-) ^b^ CEmax as measured by DCE-MRI might aid in discerning MF patients from both ET/PV patients and healthy controls **(PPV 83–85%, NPV 87%, sensitivity 88%, specificity 83–84%; Courcoutsakis et al.)** - Changes in contrast amplitude on DCE-MRI have been observed during treatment
**31-P MRS**	- Increased peaks theoretically reflect increased splenic membrane metabolism in PV/MF (da-) - Was not found to differentiate between various causes of splenomegaly (da-)
^**18**^**F-FDG and** ^**18**^**F-FLT PET/CT**	- ^18^F-FLT PET and ^18^F-FDG PET probably indirectly reflect BM fibrosis in MF and changes herein during treatment (da-) - ^18^F-FLT PET might aid in differentiating ET, PV and MF patients from healthy controls (da-) - ^18^F-FDG PET might aid in differentiating PV patients from healthy controls (da-) - Axial ^18^F-FDG uptake might correspond to prognosis in MPN ^b 18^F-FDG PET might be able to detect residual disease after stem cell transplantation in MF **(NPV 100%, PPV 86%, sensitivity 100%, specificity 83%; Derlin et al.)**, with good reproducibility of peripheral uptake rating (**interobserver and intraobserver Cohen’s k 0.95 and 1.0, respectively**)
^**99m**^**Tc-colloid and** ^**111**^**In-Cl3 scintigraphy /SPECT**	- Both tracers have been shown to reflect BM cellularity and indirectly fibrosis in MF (da-) - Increased splenic ^111^In-Cl3 uptake possibly reflects myeloid metaplasia (da-) ^b^ Tc99-colloid scintigraphy might aid in differentiating PV from secondary polycythemia (derived sensitivity 83%, derived specificity 100%; Rudberg et al)* - Both tracers might aid in differentiating early PV from MF (da-), but discriminative power will probably decrease in later PV stages given the overlap in described uptake patterns
**Labeled RBC scintigraphy**	- Increased fixation theoretically reflects splenic hypervascularization−/cellularity in MF and PV (da-) - Was not found to differentiate between various hematological diseases (da-)
**Scintigraphy – other**	- Skeletal ^52^Fe distribution might correspond to prognosis in MF - Axial ^99m^Tc-AGAb uptake might aid in discerning MF patients from controls (da-) - Skeletal ^99m^Tc-LDL uptake might reflect hematopoietic/macrophage cellularity in MF and PV (da-) - ^99m^Tc-MDP might be able to detect bone new formation in MF and PV (da-)
**Radionuclide - blood flow**^a^	- Seemed to indicate BM hypervascularization in PV and MF compared with healthy controls (da-)
**Thermo-graphy**	- Possibly indirectly reflects BM vascularization in MF (da-) - Might aid in discerning MF patients from healthy controls (da-)

*BM* bone marrow, *CEmax* maximum contrast enhancement, *CSI* chemical shift imaging, *CT* computed tomography, *(D) CE* (dynamic) contrast enhanced, *DXA* dual-energy X-ray absorptiometry, *ET* essential thrombocytosis, *HR-pQCT* high-resolution peripheral quantitative CT, *MF* myelofibrosis, *MRI* magnetic resonance imaging, *NPV* negative predictive value, *OS* overall survival, *PET* positron-emission tomography, *PPV* positive predictive value, *PV* polycythemia vera, *RBC* red blood cell, *RES* reticuloendothelial system, *sign* significant, *STIR* short tau inversion recovery

It is indicated in brackets whether diagnostic accuracy (da) was reported (*parameters; author*), not reported (−) or derived from published data (*derived parameters; author*)^b^

^a^ blood flow measurements using radionuclide imaging as described in the main text

^b^ diagnostic accuracy was not primarily reported by the authors, but was estimated from published data

#### General summary of imaging findings

##### Bone marrow – hematopoietic compartment

Evaluation of the hematopoietic compartment was done using MRI, scintigraphy, SPECT, or PET scanning. Table 2 lists commonly used MRI sequences and their corresponding tissue signal intensities. Table 3 provides an overview of used radiopharmaceuticals.

**Table 2:** MRI sequences and tissue signal intensities [3, 8]

	T1-weighted spin-echo (representing the sum of in-phase signals of fat and water)	STIR (enhances the difference in longitudinal relaxation of fat and water)	Chemical shift imaging (out-of-phase images visualizing the absolute difference between water and fat signals)
**Fat**	High	Low	High
**Water**	Low	High	High
**Cellular marrow** (≈ 40% fat, 40% water, 20% protein)	Intermediate (higher than muscle)	Intermediate (higher than muscle)	Low
**Fatty marrow** (≈ 80% fat, 15% water, 5% protein)	High	Low	High
**Fibrosis**	Low	Low	Low

*MRI* magnetic resonance imaging, *STIR* short tau inversion recovery

In general, marrow characteristics are mainly based on its fat and water content, since bone lacks mobile protons [76]. Description of brightness is relative, as compared to the signal intensity of adjacent muscle or intervertebral disc

**Table 3:** Overview of radiopharmaceuticals

Tracer	Intended use^**a**^
^**18**^**F-fluorodeoxyglucose** (^18^F-FDG)	Visualization of increased glucose metabolism / inflammatory activity [36]
^**18**^**F-3′-fluoro-3′-deoxy-L-thymidine** (^18^F-FLT)	Visualization of DNA-synthesis / cellular proliferation [37]
***Labeled colloids*** ^*99m*^*Tc,* ^*198*^*Au,* ^*113m*^*In*	Visualization of reticuloendothelial system / medullary stroma [25, 39]
^**111**^**In-Chloride** (^111^In-Cl3)	Visualization of erythropoietic activity [39]
**Labeled iron** ^*59*^*Fe,* ^*52*^*Fe*	Visualization of erythropoietic marrow [40, 45]
**Labeled red blood cells** (RBC’s) ^*99m*^*Tc or* ^*113m*^*In*	Visualization of RBC pools; heat-damaged RBC’s for assessment of spleen volume [71]
^***99m***^***Tc*-leukocytes**	Visualization of hematopoiesis [41] (standard use for detection of inflammation)
^***99m***^***Tc*-antigranulocyte antibodies** (^*99m*^*Tc*-AGAb)	Visualization of granulocyte (precursors) / granulopoeisis [46]
^***99m***^***Tc*-low density lipoprotein** (^*99m*^*Tc*-LDL)	Visualization of LDL biodistribution / catabolism [47]
^***99m***^***Tc*-methylene-diphosphonate** (^99m^Tc-MDP)	Visualization of bone matrix turnover and/or blood flow [42]
^**133**^**Xe**	Evaluation of blood flow [60] (standard use for ventilation studies)
^**15**^**O-carbon dioxide** (^15^O-CO2)	Evaluation of blood flow [61]

^a^ Concerns the intended use of the tracer as reported by the articles under review. A random choice of relevant references were appended for background reading

On T1-weighted imaging, a low signal in the axial BM was reported in only a few ET patients [19, 20], whilst low T1 signals/prolonged vertebral T1 relaxation times were more common in PV [20–24]. MR spectroscopy (MRS) indicated normal T1 values of the water resonance in a few PV patients, suggesting an increased BM water content underlying the low T1 signals. Indeed, ^18^F-FDG PET/CT and BM scintigraphy with ^111^In-Cl3, ^99m^Tc-colloid or ^198^Au-colloid (i.e. ‘reticuloendothelial scanning’) showed increased or normal axial uptake in PV with varying peripheral BM expansion, implying increased cellularity / cellular activity [25–29]. In advanced (or ‘spent phase’) PV, reported axial ^111^In-Cl3 uptake remained normal or high, whilst ^99m^Tc-colloid uptake was more often decreased [27, 30]. Normal uptake on reticuloendothelial scanning was seen in patients after chemotherapeutic treatment [27, 28].

In MF, a low T1 signal in the axial BM was seen in all patients, indicating a decrease in fatty marrow [8, 19, 20, 23, 24, 31–33]. This signal distribution was homogeneous or patchy and sometimes extended into the appendicular skeleton [19, 23]. Dixon quantitatively confirmed markedly decreased fat fractions in a few patients [33, 34]. Diffusion-weighted MRI showed increased BM signals in two patients [35]. On short-tau inversion recovery (STIR) images, skeletal signals ranged from high tot low [8, 31, 32], and were presumed to reflect the degree of cellularity [31]. In analogy to PV, ^18^F-FDG PET/CT showed high axial BM uptake with variable degrees of peripheral BM uptake in MF patients [36]. In contrast, distribution patterns on ^18^F-FLT PET/CT ranged from near-normal to decreased axial uptake with peripheral BM expansion [35, 37, 38]. Axial uptake of ^111^In-Cl3 and ^99m^Tc-colloid as detected by scintigraphy were mostly normal or low [27, 28, 30, 38–44], whilst high peripheral BM uptake was common [27, 28, 30, 38–42]. ^52^Fe scintigraphy demonstrated increased peripheral and axial BM uptake in 37% of MF patients [45]. Varying distribution patterns of ^99m^Tc-leukocytes, ^99m^Tc-LDL, and ^99m^Tc-AGAb were reported in small patient numbers [41, 46, 47].

##### Bone marrow – bone density

In PV, only non-specific skeletal changes on conventional radiography were reported, including an osteoporotic appearance and hypertrophic osteoarthritic changes [48]. On DXA and high resolution peripheral qCT (HR-pQCT), no significant differences in spinal/femoral bone mineral density (BMD) and bone geometry, microarchitecture, or strength were found between ET/PV patients and healthy controls [49].

In MF, skeletal abnormalities on radiography were seen more often, with a prevalence of increased radiodensity of 23–100% in overall populations [50–55]. Skeletal distribution was diffuse or patchy, with a predominance for the axial skeleton and proximal extremities [50–52, 54]. A decreased skeletal radiodensity was noted in 7.5–43% of patients [50, 51]. Studies on DXA yielded conflicting results. Whilst a larger case-control study found normal femoral BMDs and non-significantly increased spinal BMDs in MF patients compared with controls [56], two smaller studies did describe increased femoral BMDs in the majority of patients [55, 57]. Increased lumbar BMD was confirmed on qCT in four of the latter patients [55]. On HR-pQCT, high trabecular numbers and bone mass were described in MF compared with healthy controls, albeit not statistically significant [56]. Other clues for increased bone formation in a few MF patients were found on fat-suppressed T2-weighted MRI (high signals of the metaphyses) [55] and ^99m^Tc-MDP scintigraphy (increased uptake) [42].

##### Bone marrow – vascularity

DCE-MRI demonstrated no significant differences in perfusion parameters between a group of ET/PV patients and controls [58]. However, increased peak contrast enhancement (CE) ratios in water+fat fractions were demonstrated in two other PV patients, with normal peak CE ratios in the water fraction, thus suggesting decreased fat fractions with normal vascular density [59].

In MF, a significant increase in MR signal ratio between BM and intervertebral disk was demonstrated in several patients after intravenous contrast administration, in accordance with increased microvessel density on BM biopsies [32]. On DCE-MRI, significantly increased contrast wash-in (WIN), maximum CE (CEmax) and WIN/time-to-maximum slope were reported [58]. Infrared thermography indicated a 1.5–4 °C temperature difference between skin overlying bone and adjacent skin in MF patients with histopathological evidence of increased blood vessel endothelium, but not in controls [42]. Additional evidence for increased skeletal blood flow in a small number of PV and MF patients came from studies that used PET (with ^15^O-CO2) or scintigraphy (with ^99m^Tc-MDP, ^18^F, or ^133^Xe) [42, 60–63]. Conflicting results regarding the correlation between blood flow and BM cellularity were reported [60, 61].

##### Liver and spleen

On conventional ultrasound, the reported echotexture of (enlarged) livers and spleens in MF patients was usually normal, despite proven myeloid metaplasia in a few cases [64, 65]. Focal splenic and hepatic lesions (hypo−/anechoic or hyperechoic) were reported in 16 and 23% of patients during follow-up, respectively. Both myeloid metaplasia and metastases were described underlying these lesions [64]. On transient elastography, slightly elevated median and mean hepatic stiffness values were reported in MF patients compared with known reference values, albeit with a wide range [65–67]. Splenic stiffness values were markedly higher compared with a group of healthy controls [65, 66]. Shear wave elastography results were slightly different compared to transient elastography, with correlation coefficients of 0.78 and 0.21 for splenic and hepatic imaging, respectively [65]. Splenic tissue characterization via sound speed measurements and attenuation studies indicated no significant differences between MF patients and healthy controls [68–70], although sound speed was significantly lower in patients with higher BM fibrosis grades [69]. Regarding the etiology of splenomegaly, a study using ^99m^Tc-red blood cell (RBC) and ^113m^In-heat-damaged RBC scintigraphy demonstrated a significant correlation between splenic size and hypervascularization in both MF and PV, whilst a correlation with hypercellularity was only seen in MF [71]. In line with these findings, variably increased splenic uptake of ^18^F-FDG, ^18^F-FLT and ^111^In-Cl3 has been demonstrated in MF, often combined with low axial and/or high peripheral BM uptake [27, 28, 30, 35–39]. Lastly, increased splenic membrane metabolism was suspected in one PV and three MF patients because of increased phosphomonoester/Pi and phosphomonoester/B-ATP ratios as measured by splenic 31-phosphor MRS [72].

#### The value of imaging in diagnosis

##### Abnormalities in the hematopoietic compartment

Five studies described general correlations between imaging results and histopathological findings. Measures of diagnostic accuracy were reported by one study, and could be derived from three additional studies.

In a case-control study including 20 MF and 18 ET patients, the vertebral T1 signal correlated significantly with the histopathological fat fraction [19], but cut-off values were not defined. Another study including one PV and three ET patients demonstrated that the vertebral/spinal cord T1 signal ratio can estimate BM cellularity in patients with homogeneous disease distribution and reciprocity between cellular and adipose fractions. For the formula ‘BM cellularity(%) = 131.2–(79.6xMR ratio)’, the mean difference between observed and predicted BM cellularity was 5.6% (SD 4.0) [20]. Since such reciprocity is not present in MF, other studies have added STIR to T1-weighted imaging to differentiate between fibrosis and hypercellularity. However, in a series of 13 MF patients, T1-weighted MRI/STIR patterns did not correlate with BM biopsy results [31]. Of note, both scan results and BM histopathology were evaluated on 3-point scale, thus nuances may have been lost.

In three separate studies including 35, 15 and 55 MF patients, BM fibrosis grade inversely correlated to axial ^18^F-FDG SUVmax, spinal/proximal limb ^18^F-FLT SUVmax and skeletal ^111^In-Cl3 uptake, respectively [36, 38, 43]. However, specific values per fibrosis grade were not reported and the degree of BM cellularity was not evaluated in the first two studies. The study using ^111^In-Cl3 did report a significant correlation between skeletal uptake and BM cellularity [43], which was endorsed by three smaller studies [40, 41, 44] but refuted in another [30]. The correlation between ^99m^Tc-colloid uptake and BM findings was weaker [43]. Since ^99m^Tc-colloid uptake was generally lower than ^111^In-Cl3 uptake, and often decreased earlier in advanced disease, this tracer was presumed to reflect changes in stromal architecture in addition to hematopoietic cells [27, 30, 38, 39, 43].

Of note, reproducibility testing of the rating of peripheral BM uptake has only been performed for ^18^F-FDG PET/CT, which results in an interobserver and intraobserver Cohen’s *k* of 0.95 and 1.0, respectively [36].

One consecutive case series found a significant correlation between BMD as measured by DXA and both the histopathologic stadium and histomorphometric bone volume [57]. Of note, the sample size was small, including only three patients with histopathological osteosclerosis.

In a series of 108 consecutive MF patients, splenic stiffness correlated with BM fibrosis, with an AUROC of 0.79 for diagnosis of MF grades 2–3 [66]. Diagnostic performance improved by combining splenic stiffness with additional variables (LDH plus IPSS score) into a diagnostic model. At the optimal cut-off points, the reported accuracy hereof was 97% for identifying MF grades 0–1 and 95% for MF grades 2–3. Remarkably, this final model differed from a previous version which was presented as a conference abstract, due to different outcomes from multivariate analyses [73]. Also, it was unclear how many patients had been excluded and whether index and reference tests were evaluated in a blinded manner. No threshold was pre-specified and TE results were indeterminate in 18.5% of patients. The risk of bias was deemed high (Table 4) [66].

**Table 4:**
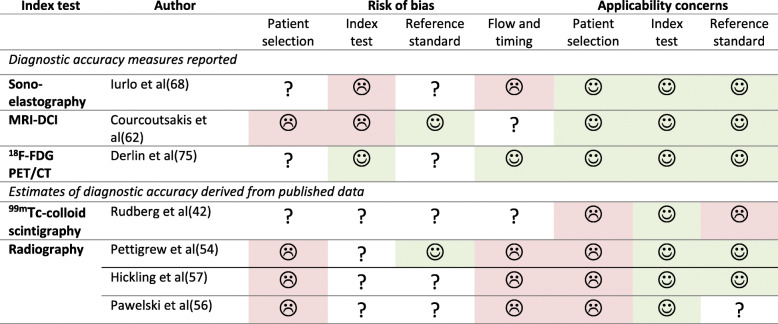
Assessment of diagnostic accuracy studies according to QUADAS-2

Three studies compared radiography with histopathological results. Sclerosis was described in 60–100% of patients with histopathological osteo (myelo) sclerosis (*n* = 59) [50, 52, 53], in 39–57% of patients with myelofibrosis without osteosclerosis (*n* = 24) [50, 52], and in none of the six reported patients with hypercellular disease [50]. In one study, radiological sclerosis correlated with the histopathological percentage of bone, but not fibrous tissue [50]. Based on these studies, we derived a sensitivity of 60–100% and a specificity of 43–61% for the detection of histopathological osteosclerosis. Of note, biopsies were not necessarily taken from depicted areas, which might have influenced results. Also, risk of bias and applicability concerns across these studies were high (Table 4), due to outdated diagnostic criteria and uncertainties in patient inclusion, the intended use for the index test, and the time interval between index and reference tests (> 1 year in one study). In two studies it was unclear whether index and reference tests were evaluated in a blinded manner. Lastly, one study reported multiple observations in 5/23 patients, which we treated as independent measurements [50].

##### Differentiation of ET, PV, and MF

Two studies compared imaging results in the MPN subtypes without calculating diagnostic accuracy. One study did report measures of diagnostic accuracy, and we were able to derive them from another.

One case-control study demonstrated a significantly decreased vertebral T1 signal and histopathological fat fraction in 20 MF patients, whereas in 18 ET patients both parameters were near-normal. The femoral signal intensity was also significantly lower in MF [19]. Of note, disease severity and degree of BM cellularity were not reported. Another study found similar axial ^111^In-Cl3 uptake amongst 55 MF and 15 PV patients, with only slightly lower ^99m^Tc-colloid uptake in MF [43]. MF patients did show higher splenic ^111^In-Cl3 uptake, but cut-off values were not defined.

In a case-control study including 12 MF, 6 ET and 6 PV patients, DCE-MRI was used to determine BM perfusion parameters (as described above). CEmax appeared most accurate in distinguishing MF from ET/PV patients and healthy controls, with respective positive and negative predictive values of 83–85 and 87% [58]. Of note, the threshold was not pre-specified and disease stages were unclear. Also, patient selection was not clearly described and it was unknown whether interpretation of the index test was done without knowledge of the reference test. Combined, this results in a considerable risk of bias (Table 4).

Lastly, one study performed ^99m^Tc-albumin scintigraphy in 37 patients with polycythemia. High pelvic ^99m^Tc-albumin fixation was shown to discern 19 PV patients from those with secondary polycythemia [29]. Although not primarily reported, we derived a sensitivity and specificity of 84 and 100% at the pre-specified cut-off value of 150kcnt/180 s. However, diagnostic criteria were outdated and patient inclusion was not clearly described. It was unclear whether interpretation of index and reference tests were done without knowledge of the other. Therefore, the risk of bias is largely unclear and applicability concerns exist (Table 4).

#### The value of imaging in prognostication

Three studies described a correlation between of imaging results and prognosis in MF, of which only one quantified the extent.

In an explorative study including 13 MF patients, patterns on combined T1-weighted MRI and STIR imaging correlated significantly with prognostic staging [31], with the lowest femoral and axial T1 and STIR signals occurring in high risk patients. Of note, this prognostic staging was based on part of an old scoring system and actual survival data were not provided. Another study including 50 MF patients stated that death was more frequent when axial ^99m^Tc-colloid and ^111^In-Cl3 BM uptake were very poor and peripheral extension was weak, but no quantification hereof was provided [30]. Lastly, a study including 59 MF patients described a higher probability of 3-year survival in MF patients without increased peripheral ^52^Fe BM uptake versus those with increased uptake (66% vs 33%), but this difference was not statistically significant [45]. Mean duration of follow-up and number of events were not reported.

#### The value of imaging in follow-up monitoring

Seven studies described results of repeated imaging during follow-up and/or treatment of MF, of which only one presented diagnostic accuracy results.

One study evaluated splenic transient elastography before and after treatment with ruxolitinib in a subgroup of three MF patients [66]. All three patients showed a decrease in splenic stiffness (mean decrease 13 kPa), along with a decrease in spleen size. Of note, the reason for follow-up evaluations in these patients and therapy duration were not reported [66]. In another study, a subgroup of nine MF patients underwent repeated splenic sound speed measurements. An increase in sound speed was found in eight MF patients treated with chemotherapy (at 5–80 weeks after start of bulsulphan or 6-thioguanine), which correlated with a decrease in BM fibrosis and splenomegaly [69]. In contrast, the one MF patient who did not receive chemotherapy showed stable splenic sound speed after 10 months. Again, the reason for follow-up evaluations in these specific patients was unclear. One study described results of repeated DXA scans in two out of four included MF patients with known osteosclerosis. Both patients showed a further increase in lumbar BMD after 2–4 years, whilst femoral BMD increased in only one out of two [55]. No clinical follow-up details were provided.

One study performed sequential T1-weighted MRI/STIR and BM biopsies before allo-SCT in 35 MF patients, with planned repeated measurements at two later time points [8]. Since many patients declined repeat examinations, follow-up scans were available for 21 and 10 patients after 3 months and 1–2 years, respectively. A (partial) normalization of the T1 and STIR signals occurred in 19/21 patients. Changes usually started in the femora, and later progressed to the pelvis and vertebrae. The most pronounced changes were seen after more than one year post-allo-SCT. Histopathological fibrosis reduction was also seen in 18/19 patients, but discrepancies regarding the degree of reduction often existed compared to MRI results. These discrepancies were attributed to the effects of inhomogeneous disease, since MRI demonstrated biopsy needle tracks in non-representative areas (i.e. ‘sampling error’). Of note, the MRI scoring system for fibrosis was not explained in detail, thus limiting its external use. Also, selection bias might have occurred due to the high study drop-out rate. In a study using Dixon, four MF patients were evaluated before start of ruxolitinib treatment, and after 1–2 months, 3–5 months, and 6–10 months of treatment, respectively. Two out of four patients showed increasing pelvic and femoral fat fractions over time, along with a normalization of BM cellularity but with stable splenomegaly [34]. The most pronounced increases in fat fractions occurred after 5 months or longer. Interestingly, the two patients with stable fat fractions did show a significant decrease in splenomegaly. Unfortunately, no follow-up BM biopsies were available in these cases. One study performed repeated ^99m^Tc-colloid and ^111^In-Cl3 scintigraphy in a subgroup of six MF patients [43]. Three untreated patients showed a decrease in axial uptake with an increase of splenic ^111^In-Cl3 uptake, whilst two patients treated with hydroxyurea showed a reversed pattern. However, the reasons for follow-up examinations, duration of follow-up and baseline results were not provided. One pilot study retrospectively evaluated 12 MF patients in whom ^18^F-FDG PET scans and BM biopsies were available both before and after allo-SCT (range: 100–442 days). A decrease in SUVmax_bm post-transplantation was found to correspond to a decrease in fibrosis grade [74]. Using the IWG-MRT criteria as the reference standard, the sensitivity of ^18^F-FDG PET for detecting residual disease was 100%, with a specificity of 83% and negative and positive predictive values of 100 and 86%, respectively [74]. Risk of bias seems low, although the initial indication for imaging and patient inclusion criteria were unclear, and uncertainty regarding the blinded interpretation of the reference test exists (Table 4).

## Discussion

A wide variety of imaging methods used in the evaluation of the JAK2/CALR/MPL mutation-related MPNs was identified via our systematic literature search. Most studies were explorative, and a minor subset reported correlations of imaging results to histopathological findings, disease severity, or treatment response. Diagnostic accuracy could be extracted or derived from seven studies. Nonetheless, findings can give direction to clinical practice and future research.

Regarding diagnosis of BM cellularity, T1-weighted MRI might be sufficient in ET and PV, although larger studies are required to confirm the accuracy of the formula defined by Rozman et al.. In MF, both T1-weighted MRI and Dixon seem to represent BM fat content, but they do not differentiate between hypercellularity and fibrosis. The addition of STIR might facilitate this differentiation, but the value of this technique alone could not be concluded based on published data. Nevertheless, the potential prognostic value of combined T1-weighted MRI/STIR imaging, along with their seemingly accurate reflection of response after allo-SCT, warrant further research in this field. Importantly, uniform grading of distribution patterns is required. Since Dixon offers the possibility of quantifying fat fractions, it forms an attractive alternative.

^18^F-FDG PET/CT, ^18^F-FLT PET/CT, and BM scintigraphy using ^111^In-Cl3- and ^99m^Tc-colloid form reasonable alternatives for characterization of the hematopoietic compartment in PV and/or MF. Interestingly, although these techniques are meant to visualize cellularity/cellular activity, a correlation between uptake and BM cellularity was only demonstrated for ^111^In-Cl3 and ^99m^Tc-colloid (with unclear diagnostic accuracy). However, ^18^F-FDG, ^18^F-FLT-, ^111^In-Cl3-, and ^99m^Tc-colloid uptake were all inversely correlated with BM fibrosis grade in MF, providing potential value for disease monitoring. Indeed, in the well conducted study by Derlin et al., diagnostic accuracy of ^18^F-FDG PET/CT regarding residual disease after treatment of MF was high. Furthermore, reproducibility of this technique was demonstrated in a separate study. However, confirmation of these results in a second study is necessary before incorporation into clinical practice, given the small sample size.

Although hybrid imaging with ^111^In-Cl3-, and ^99m^Tc-colloid also showed potential value in follow-up monitoring and prognostication, this technique has practical drawbacks. It requires two imaging acquisitions, 48 h apart, and ^99m^Tc-colloid scintigraphy is not useful for splenic evaluation. Furthermore, quantitative analysis of ^18^F-FLT PET images has proven to be easier [38]. Lastly, evidence on scintigraphy combined with other tracers (^99m^Tc-LDL, ^99m^Tc-leukocytes, ^99m^Tc-AGAb) is scarce.

Regarding evaluation of osteosclerosis, we found no evidence for a standard application of conventional radiography in PV or MF. Despite the risk of bias in most studies, the diagnostic accuracy for the diagnosis of osteosclerosis is insufficient. DXA might form an interesting alternative in MF, since it showed a correlation to histopathological bone volume. However, its discriminative ability in early disease stages is questionable. Data on HR-pQCT, qCT and scintigraphy using bone-seeking tracers are limited. Theoretically, the sensitivity of HR-pQCT could be insufficient in early disease stages, given the origination of abnormalities in the axial skeleton. Bone scintigraphy might provide more functional information on osteoblastic activity. In further evaluation hereof, the effect of BM blood flow on uptake of bone tracers should be taken into account. Several methods for evaluation of the latter seem promising, including DCE-MRI, skin thermography, scintigraphy and PET, but more research is needed. Although the predictive value of CEmax for differentiating MF and ET/PV patients seemed reasonable, this semi-quantitative outcome measure is sensitive to variations between patients and acquisition protocols, which complicates the definition of cut-off values and comparison between patients. Alternatively, quantitative parameters could be used, although these require the acquisition of a patient-derived arterial input function [75].

Regarding evaluation of splenomegaly, conventional ultrasound is often used in clinical practice. However, beyond size measurements this technique will likely not aid in diagnosis of MF, although abnormalities might warrant further evaluation. Also, splenic attenuation studies seem to be of limited value and data on 31-phosphor MRS are too scarce to recommend its use. Splenic transient elastography and sound speed measurements might indirectly reflect BM fibrosis grade in MF, and changes herein during treatment, but several concerns exist. Firstly, reliable measurements were not feasible in a substantial part of patients and splenic stiffness might be influenced by concurrent thrombotic disease. Furthermore, the study by Iurlo et al. carried a substantial risk of bias. Lastly, since the decreases in splenic stiffness and splenic sound speed during treatment coincided with a decrease in spleen size, the added value of these measurements is unclear.

## Conclusions

Apart from the possible use of T1-weighted imaging to estimate BM cellularity, no techniques were described to aid in diagnosis, prognostication or follow-up of ET. In PV, ^18^F-FDG PET/CT and BM scintigraphy using ^111^In-Cl3- and ^99m^Tc-colloid might reflect disease activity/severity, but diagnostic accuracy hereof is unknown.

In MF, T1-weighted MRI, STIR and Dixon are promising techniques for the evaluation and follow-up of BM fat content (and indirectly hypercellularity and fibrosis), but diagnostic accuracy is unknown. Results of ^18^F-FDG PET/CT, ^18^F-FLT PET/CT, and BM scintigraphy using ^111^In-Cl3- and ^99m^Tc-colloid all correlated to BM fibrosis, thus providing potential value for disease monitoring. However, diagnostic accuracy was only determined for ^18^F-FDG PET/CT, which seemed to be high after allo-SCT. Conventional radiography seems insufficiently accurate for the diagnosis of osteosclerosis. Alternative techniques, including DXA, HR-pQCT and bone scintigraphy require further evaluation. Multiple studies suggested increased BM blood flow in MF, including DCE-MRI. Although this technique seemed to reasonably distinguish MF patients from ET/PV patients, it has multiple drawbacks. Finally, splenic transient elastography combined with clinical parameters might indirectly reflect BM fibrosis in MF, but the added value during follow-up monitoring is questionable.

Although evaluation of diagnostic accuracy might be complicated by imperfections in the reference standard (e.g. sampling error), many studies also showed methodological limitations. We hope that future investigators will improve study designs, including standardized interpretation of results with blinded interpreters, a clear reference standard, inclusion of consecutive patients and a systematic presentation of results (preferably using two-by-two tables and pre-specified cut-off values). In addition, it is essential to examine the prognostic relevance of imaging appearances in different disease stages. This will require studies with longer follow-up and/or comparison to existing prognostic scoring systems. Lastly, in search for a new diagnostic test, reproducibility, safety, availability and costs should be taken into account. We hope that our current review can aid future researchers in the choice of imaging techniques and study designs.

## Supplementary Information

**Additional file 1.** Supplementary information: search strategies and overview of included articles.

## Data Availability

The datasets used and/or analyzed during the current study are available from the corresponding author on reasonable request.

## Abbreviations

BM: Bone marrow
BMD: Bone mineral density
CEmax: Maximum contrast enhancement
CSI: Chemical shift imaging
CT: Computed tomography
DCE-MRI: Dynamic contrast-enhanced magnetic resonance imaging
DXA: Dual-energy X-ray absorptiometry
ET: Essential thrombocythemia
^18^F-FDG: ^18^F-fluorodeoxyglucose
^18^F-FLT: ^18^F-fluorothymidine
HR-pQCT: High-resolution peripheral quantitative computed tomography
IPSS: International Prognostic Scoring System
MF: Myelofibrosis
MPNs: Myeloproliferative neoplasms
MRI: Magnetic resonance imaging
MRS: Magnetic resonance spectroscopy
PDE: Phoshodiester
PET: Positron emission tomography
PME: Phosphomonoester
PRISMA: Preferred Reporting Items for Systematic Reviews and Meta-Analysis
PV: Polycythemia vera
RBC: Red blood cell
SCT: Stem cell transplantation
SPECT: Single photon emission computed tomography
STIR: Short tau inversion recovery
SUV: Standardized uptake value
WIN: Wash-in
