# Supplementary material for: The value of bone marrow, liver, and spleen imaging in diagnosis, prognostication, and follow-up monitoring of myeloproliferative neoplasms: a systematic review
Source: Cancer Imaging. 2021 Apr 20;21:36. doi: 10.1186/s40644-021-00405-7 (PMC8056651; doi:10.1186/s40644-021-00405-7)
Supplement: Supplementary file 1 — Additional file 1. Supplementary information: search strategies and overview of included articles. [file 40644_2021_405_MOESM1_ESM.docx]

**Supplemental material**

**Index**

1. Supplemental methods: search strategies
2. Supplemental table: overview of included articles (+ references)
3. **Supplemental methods: search strategies**

*PubMed (2020 March 26^th^)*

| **#** | **Query** | **Results** |
| --- | --- | --- |
| #14 | #5 OR #7 OR #9 OR #11 OR #13 | 2306 |
| #13 | #3 AND #12 | 1618 |
| #12 | "Ultrasonography"[Mesh] OR "diagnostic imaging" [Subheading] OR ultraso*[tw] OR sonograph*[tw] OR echograph*[tw] OR echotomograph*[tw] | 1506148 |
| #11 | #3 AND #10 | 544 |
| #10 | "Magnetic Resonance Imaging"[Mesh] OR MRI[tw] OR MRIs[tw] OR NMR[tw] OR NMRs[tw] OR fMRI[tw] OR “magnetic resonance”[tw] OR “MR imaging”[tw] OR “nuclear magnetic”[tw] OR (tomogra*[tw] AND (MR[tw] OR “proton spin”[tw])) OR ((“chemical shift”[tw] OR “chemical shifts”[tw]) AND imaging*[tw]) OR “Magnetization Transfer”[tw] OR “Magnetisation Transfer”[tw] OR “resonance magnetic”[tw] OR MR imag*[tw] | 862888 |
| #9 | #3 AND #8 | 813 |
| #8 | "Tomography, X-Ray Computed"[Mesh] OR (compute*[tw] AND tomograph*[tw]) OR ((CAT[tw] OR CT[tw]) AND (scan[tw] OR scans[tw] OR scanning[tw] OR X-ray[tw] OR X-rays[tw])) | 649787 |
| #7 | #3 AND #6 | 20 |
| #6 | (“Whole body imaging”[Mesh] OR "whole body"[tw]) AND ("X-Rays"[Mesh] OR "Radiography"[Mesh] OR "diagnostic imaging" [Subheading] OR “x-ray”[tw] OR “x-rays”[tw] OR x-radiation*[tw] OR roentgen*[tw] OR radiography[tw] OR radiographic[tw] OR radiology[tw] OR radiogram[tw] OR radioimaging[tw]) | 14959 |
| #5 | #3 AND #4 | 135 |
| #4 | "Tomography, Emission-Computed"[Mesh] OR pet[tw] OR pet/*[tw] OR petscan*[tw] OR (emission[tw] AND (tomograph [tw] OR tomographs [tw] OR tomographic*[tw] OR tomography[tw] OR tomographies[tw] OR scan[tw])) | 166935 |
| #3 | #1 OR #2 | 35117 |
| #2 | Myeloproliferati*[tiab] OR Polycythemia*[tiab] OR Polycythaemia*[tiab] OR Erythremia*[tiab] OR Erythraemia*[tiab] OR Myelofibrosis[tiab] OR Myelofibroses[tiab] OR Myelosclerosis[tiab] OR Myeloscleroses[tiab] OR Thrombocythemia*[tiab] OR Thrombocythaemia*[tiab] OR Thrombocytosis[tiab] OR Thrombocytoses[tiab] OR Osler-Vaquez[tiab] OR Bone marrow fibrosis[tiab] OR Bone marrow fibroses[tiab] OR Myeloid metaplasia*[tiab] OR Nonleukemic myelosis[tiab] OR Nonleukemic myeloses[tiab] OR Non-leukemic myelosis[tiab] OR Non-leukemic myeloses[tiab] | 29027 |
| #1 | "Myeloproliferative Disorders"[Mesh:NoExp] OR "Polycythemia Vera"[Mesh] OR "Primary Myelofibrosis"[Mesh] OR "Thrombocythemia, Essential"[Mesh] OR "Polycythemia"[Mesh] | 23176 |

*Embase.com (2020 March 26^th^)*

| **#** | **Query** | **Results** |
| --- | --- | --- |
| #14 | #5 OR #7 OR #9 OR #11 OR #13 | **4391** |
| #13 | #3 AND #12 | **2272** |
| #12 | 'echography'/exp OR ultraso*:ab,ti OR sonograph*:ab,ti OR echograph*:ab,ti OR echotomograph*:ab,ti | **1080660** |
| #11 | #3 AND #10 | **543** |
| #10 | ('nuclear magnetic resonance'/exp OR 'nuclear magnetic resonance imaging'/exp OR mri:ab,ti OR mris:ab,ti OR nmr:ab,ti OR nmrs:ab,ti OR fmri:ab,ti OR 'magnetic resonance':ab,ti OR 'mr imaging':ab,ti OR 'nuclear magnetic':ab,ti OR (tomogra*:ab,ti AND (mr:ab,ti OR 'proton spin':ab,ti)) OR (('chemical shift':ab,ti OR 'chemical shifts':ab,ti) AND imaging*:ab,ti) OR 'magnetization transfer':ab,ti OR 'magnetisation transfer':ab,ti OR 'resonance magnetic':ab,ti OR mr) AND imag*:ab,ti | **552626** |
| #9 | #3 AND #8 | **2339** |
| #8 | 'computer assisted tomography'/exp OR (compute*:ab,ti AND tomograph*:ab,ti) OR ((cat:ab,ti OR ct:ab,ti) AND (scan:ab,ti OR scans:ab,ti OR scanning:ab,ti OR 'x ray':ab,ti OR 'x rays':ab,ti)) | **1155750** |
| #7 | #3 AND #6 | **14** |
| #6 | ('whole body imaging'/exp OR 'whole body':ab,ti) AND ('x-ray'/exp OR 'radiography'/exp OR 'x-ray':ab,ti OR 'x-rays':ab,ti OR 'x radiation':ab,ti OR 'x radiations':ab,ti OR roentgen*:ab,ti OR radiography:ab,ti OR radiographic:ab,ti OR radiology:ab,ti OR radiogram:ab,ti OR radioimaging:ab,ti) | **9940** |
| #5 | #3 AND #4 | **497** |
| #4 | pet:de,ab,ti OR 'emission tomography'/exp OR (emission:de,ab,ti AND (tomograph:de,ab,ti OR tomographs:de,ab,ti OR tomographic*:de,ab,ti OR tomography:de,ab,ti OR tomographies:de,ab,ti OR scan:de,ab,ti)) | **306507** |
| #3 | #1 OR #2 | **51376** |
| #2 | myeloproliferati*:ab,ti OR polycythemia*:ab,ti OR polycythaemia*:ab,ti OR erythremia*:ab,ti OR erythraemia*:ab,ti OR myelofibrosis:ab,ti OR myelofibroses:ab,ti OR myelosclerosis:ab,ti OR myeloscleroses:ab,ti OR thrombocythemia*:ab,ti OR thrombocythaemia*:ab,ti OR thrombocytosis:ab,ti OR thrombocytoses:ab,ti OR 'osler-vaquez':ab,ti OR 'bone marrow fibrosis':ab,ti OR 'bone marrow fibroses':ab,ti OR ((myeloid NEXT/1 metaplasia*):ab,ti) OR 'nonleukemic myelosis':ab,ti OR 'nonleukemic myeloses':ab,ti OR 'non-leukemic myelosis':ab,ti OR 'non-leukemic myeloses':ab,ti | **41548** |
| #1 | 'myeloproliferative disorder'/de OR 'polycythemia vera'/exp OR 'myeloid metaplasia'/exp OR 'thrombocythemia'/exp OR 'polycythemia'/exp | **33130** |

*Cochrane Library via Wiley (2020 March 26^th^)*

| **#** | **Query** | **Results** |
| --- | --- | --- |
| #12 | #3 or #5 or #7 or #9 or #11 | **138** |
| #11 | #1 and #10 | **21** |
| #10 | ultraso*:ti,ab,kw or sonograph*:ti,ab,kw or echograph*:ti,ab,kw or echotomograph*:ti,ab,kw | **41453** |
| #9 | #1 and #8 | **84** |
| #8 | "Magnetic Resonance Imaging":ti,ab,kw or MRI:ti,ab,kw or MRIs:ti,ab,kw or NMR:ti,ab,kw or NMRs:ti,ab,kw or fMRI:ti,ab,kw or "magnetic resonance":ti,ab,kw or "MR imaging":ti,ab,kw or "nuclear magnetic":ti,ab,kw or (tomogra*:ti,ab,kw and (MR:ti,ab,kw or "proton spin":ti,ab,kw)) or (("chemical shift":ti,ab,kw or "chemical shifts":ti,ab,kw) and imaging*:ti,ab,kw) or "Magnetization Transfer":ti,ab,kw or "Magnetisation Transfer":ti,ab,kw or "resonance magnetic":ti,ab,kw or "MR imag*":ti,ab,kw | **33676** |
| #7 | #1 and #6 | **31** |
| #6 | (compute*:ti,ab,kw and tomograph*:ti,ab,kw) or ((CAT:ti,ab,kw or CT:ti,ab,kw) and (scan:ti,ab,kw or scans:ti,ab,kw or scanning:ti,ab,kw or "X-ray":ti,ab,kw or "X-rays":ti,ab,kw)) | **22842** |
| #5 | #1 and #4 | **0** |
| #4 | "whole body":ti,ab,kw and ("x-ray":ti,ab,kw or "x-rays":ti,ab,kw or x-radiation*:ti,ab,kw or roentgen*:ti,ab,kw or radiography:ti,ab,kw or radiographic:ti,ab,kw or radiology:ti,ab,kw or radiogram:ti,ab,kw or radioimaging:ti,ab,kw) | **80** |
| #3 | #1 and #2 | **44** |
| #2 | pet:ti,ab,kw or (emission:ti,ab,kw and (tomograph:ti,ab,kw or tomographs:ti,ab,kw or tomographic*:ti,ab,kw or tomography:ti,ab,kw or tomographies:ti,ab,kw or scan:ti,ab,kw)) | **9238** |
| #1 | Myeloproliferati*:ti,ab,kw or Polycythemia*:ti,ab,kw or Polycythaemia*:ti,ab,kw or Erythremia*:ti,ab,kw or Erythraemia*:ti,ab,kw or Myelofibrosis:ti,ab,kw or Myelofibroses:ti,ab,kw or Myelosclerosis:ti,ab,kw or Myeloscleroses:ti,ab,kw or Thrombocythemia*:ti,ab,kw or Thrombocythaemia*:ti,ab,kw or Thrombocytosis:ti,ab,kw or Thrombocytoses:ti,ab,kw or "Osler-Vaquez":ti,ab,kw or "Bone marrow fibrosis":ti,ab,kw or "Bone marrow fibroses":ti,ab,kw or "Myeloid metaplasia*":ti,ab,kw or "Nonleukemic myelosis":ti,ab,kw or "Nonleukemic myeloses":ti,ab,kw or "Non-leukemic myelosis":ti,ab,kw or "Non-leukemic myeloses":ti,ab,kw | **1309** |

1. **Supplemental table. Overview of included articles**

| **First author, year of publication** | **Study design** | **Patient inclusion** | **Imaging technique** | **No. of MPN patients and controls** | **Main findings** |
| --- | --- | --- | --- | --- | --- |
| Siniluoto, 1992 [1] | Retrospective, cross-sectional | Consecutive pt. with available ultrasounds | Abdominal ultrasound | 39 MF | Global splenic/hepatic echogenicity usually normal (sporadically hypoechoic)  Focal splenic/hepatic lesions in up to 16/23%, histopathology reported in a few cases |
| Iurlo, 2015 [2] | Probably prospective?, cross-sectional, diagnostic accuracy study | Consecutive pt. | Transient elastography  ‘ Fibroscan’ (liver, spleen) | 108 MF | Median hepatic stiffness 7.1 kPa (range 3.5-19.6), median splenic stiffness 40.1kPa (range 11.8-75).  Sign. correlation splenic stiffness and BM fibrosis, diagnostic model + accuracy measures presented |
| Webb, 2015 [3] | Probably prospective?, cross-sectional, case-control | Probably consecutive pt.? | Ultrasound, transient elastography  ‘ Fibroscan’, shear wave elastography  (liver, spleen) | 9 MF, 8 healthy controls | Homogeneous liver parenchyma in all but one MF pt.  Mean hepatic stiffness on fibroscan/SWE: 7.8/10.4kPa, splenic stiffness 41.3/32.9kPa. Correlation coefficient fibroscan/SWE r=0.71 for splenic values |
| Chen, 1987 [4]  Manoharan, 1988 [5] | Probably prospective?, cross-sectional / longitudinal | Probably consecutive pt.? | Sound speed measurements (spleen) | 19 MF, 21 healthy controls | Overall no sign. differences in splenic sound speed between MF pt. and controls, but sign. lower sound speed in higher fibrosis grades. Sound speed increased in pt. with fibrosis reduction during treatment.  No measurements possible in 25% of pt. |
| Wilson, 1987 [6] | Prospective, longitudinal | Unclear | Splenic attenuation studies | 6 MF, unknown number of controls (subgroup from Chen/Manoharan) | No sign. differences in splenic attenuation between MF pt. and controls; no correlation with fibrosis |
| Farmer, 2017 [7] | Prospective, cross-sectional, case-control | Consecutive pt. without known osteoporosis | DXA  (spine, hip) | 14 ET, 31 PV, 45 healthy controls | No significant differences in BMD between ET/PV pt. and controls |
| Farmer, 2015 [8] | Prospective, cross-sectional, case-control | Consecutive pt. without known osteoporosis | DXA  (spine, hip) | 18 MF (incl. 11 with osteo-sclerosis),  17 healthy controls | No significant differences in femoral BMD between MF pt. and controls, non-statistically significant higher spinal BMD in MF |
| Diamond, 2002 [9] | Prospective, cross-sectional / longitudinal | Consecutive pt. with osteosclerosis | Radiography, DXA, qCT, MRI | 4 MF with osteosclerosis | Prevalence of sclerosis on radiography 100%  Lumbar BMD increased in all pt., further increase over time in 2/4  High signal intensity in metaphyses on fat-suppressed T2-weighted sequences |
| Mellibovsky, 2004 [10] | Prospective, cross-sectional | Consecutive pt. without other bone metabolism disorders | DXA | 9 MF (incl. 3 with osteosclerosis) | Increased BMD in 8/9 patients, correlation with histomorphometric bone volume |
| Pettigrew, 1969 [11] | Retrospective, cross-sectional | Pt. with available radiographs (probably consecutive?) | Radiography ((parts of) axial skeleton) | 23 AMM | Prevalence of skeletal abnormalities 83% (osteosclerosis 39%). Normal/porotic appearance in all pt. with hypercellular BM; sclerotic appearance in 100% of cases with osteosclerosis but also in 39% of cases with BM fibrosis |
| Takacsi-Nagy, 1975 [12] | Probably retrospective, cross-sectional? | Unclear | Radiography (regions unclear) | 60 MF | Prevalence of skeletal abnormalities ≈ 50% (sclerosis 23%, porosis 7.5%, focal abnormalities 10%, combined abnormalities 7%). No correlation between skeletal abnormalities and BM histopathology (data not shown) |
| Pawelski, 1972 [13] | Probabaly retrospective?  Scans at different time points | Unclear | Radiography (skeleton) | 30 osteo-(myelo)-sclerosis | Prevalence of skeletal abnormalities 66% (all sclerosis)  Sclerosis in 68% of pt. with BM osteosclerosis, but also in 57% of pt. with BM fibrosis |
| Hickling, 1968 [14] | Probably retrospective, cross-sectional? | Pt. with available radiographs (probably consecutive?) | Radiography (regions unclear) | 35 MF with osteosclerosis | Prevalence of sclerosis on radiography 60%  Sclerosis in 60% of pt. with BM osteosclerosis |
| Chelloul, 1976 [15] | Retrospective, cross-sectional | Pt. with available radiographys (probably consecutive?) | Radiography (skeleton) | 71 MF | Prevalence of sclerosis on radiography 34%  No abnormalities in pt. with BM hypercellularity, abnormalities in 45% of pt. with stage II-III osteosclerosis |
| Hodgson, 1946 [16] | Retrospective, cross-sectional, observational | Pt. with available radiographs (probably selected?) | Radiography (pelvis, spine, extremities) | 41 PV | Osteoporotic appearance in 33%, hypertrophic osteoarthritic changes in 12%; abnormalities were deemed uncorrelated to PV |
| Amano, 1995 [17] | Retrospective, cross-sectional, case-control | Non-consecutive | T1- and T2-weighted MRI (mandibular bone) | 1 MF, 2 PV, 45 controls without BM disease | Low T1 signal in all pt. |
| Amano, 1997 [18] | Timeline unclear, cross-sectional | Unclear | Contrast-enhanced T1-weighted MRI, STIR (spine) | 9 MF | Low T1 signal and intermediate to high STIR signals in all pt. |
| Sale, 2006 [19] | Prospective, longitudinal | Unclear | T1-weighted MRI, STIR (pelvis, spine, femora) | 35 MF | Low T1 signal and intermediate STIR signal in all pt. at baseline.  Follow-up images in 21 pt. after treatment; improvement in BM signals plus BM fibrosis in 19/21 pt, but discrepancies in degree of change. Improvement in BM signals but stable BM fibrosis in 1 pt.  Patchy disease in 14% pre-alloSCT and 67% post-alloSCT |
| Alpdogan, 1998 [20] | Timeline unclear, cross-sectional | Unclear | T1-weighted MRI, STIR (pelvis, spine, femora) | 13 MF | 3 imaging patterns: 1A: T1 signal low axial/high femur+ STIR signal diffusely high. 1B: T1 signal diffusely low, STIR signal diffusely high. 2: T1+STIR signals diffusely low  Patterns interpreted as early hypercellular, late hypercellular and fibrotic phases, but no sign. correlation to  histopathology  Sign. correlation to prognostic scoring |
| Kaplan, 1992 [21] | Retrospective, cross-sectional | Unclear | T1- and T2 weighted MRI (pelvis, spine, femora) | 10 MF, 4 PV | Low T1 signal in all MF patients and most PV patients |
| Guckel, 1990 [22] | Prospective, cross-sectional, case-control | Unclear | T1- and T2 weighted MRI, modified Dixon (pelvis, spine, femora) | 2 MF, 9 healthy controls | Significantly decreased fat fractions in MF pt. compared to controls |
| Rozman, 1997 [23] | Probably prospective?, cross-sectional | Unclear (but in- and exclusion criteria defined) | T1-weighted MRI  (spine) | 3 ET, 1 PV (total 25 pt with hematological disease) | Significant inverse correlation between BM cellularity and vertebral/spinal cord MR ratio |
| Rozman, 1999 [24] | Timeline unclear,  cross-sectional, case-control | Unclear | T1-weighted MRI | 20 MF, 18 ET | T1 signal significantly lower in MF pt compared to ET, and more femoral reconversion in MF  Sign. correlation between BM fat content and vertebral T1 signal  Sign. lower T1 signals in MF pt. with BM fibrosis vs BM hypercellularity |
| Luker, 2016 [25] | Prospective, longitudinal | Consecutive patients with indication for ruxolitinib treatment | Multiecho Dixon  (pelvis/hip) | 4 MF | Decreased fat fractions at baseline in all patients, normalization during treatment in 2/4 |
| Jensen, 1988 [26] | Timeline unclear,  cross-sectional | Unclear | T1- and T2-weighted MRI (spine) | 8 PV | Prolonged T1 relaxation times in all patients |
| Jensen, 1990 [27] | Timeline unclear,  cross-sectional, case-control | Unclear | MRI (spine; T1 relaxation times) + MRS (pelvis) | 9 PV, 21 healthy controls | T1 relaxation times significantly prolonged in PV pt. compared to controls, with normal water resonance on MRS |
| Courcoutsakis, 2012 [28] | Prospective, cross-sectional, case-control, diagnostic accuracy study | Unclear | DCE-MRI (spine) | 12 MF, 6 ET, 6 PV, 12 controls with lumbago | Significantly different perfusion parameters in MF pt. compared to controls and ET/PV pt. |
| Katsuya, 2000 [29] | Probably prospective?, cross-sectional | Unclear | DCE-MRI  (spine) | 2 PV, 30 healthy controls | Increased peak CE ratios in water+fat fractions in PV pt. compared to controls, with similar peak CE ratios in water fractions |
| Tsujikawa, 2019 [30] | Retrospective, cross-sectional | Unclear | MRI DWI, ^18^F-FLT PET/CT | 2 MF | Diffusely increased signal on DWI in both pt.  Variable axial ^18^F-FLT uptake with distinct peripheral extension and splenic uptake in both pt. |
| Smith, 1989 [31] | Timeline unclear,  cross-sectional, case-control | Unclear | ^31^P-MRS | 3 MF, 1 PV, 6 healthy controls | Increased PME/Pi and PME/B-ATP peaks in MF/PV pt. compared to controls |
| Derlin, 2015 [32] | Retrospective, cross-sectional | Pt. referred for allo-SCT with available scans, probably consecutive? | ^18^F-FDG PET/CT | 30 MF | Increased axial SUVmean pre-allo-SCT in all pt. with variable peripheral extension. Uptake correlated to disease duration, fibrosis grade |
| Derlin, 2016 [33] | Retrospective, cross-sectional | Subgroup of pt. from Derlin 2015 with available follow-up scans, probably selected? | ^18^F-FDG PET/CT | 12 MF | Pre allo-SCT increased uptake in all pt.  PET normalization in 5/6 pt. with CR post allo-SCT; residual disease detected in 6/6 pt. without CR  Sign. correlation between decrease in lumbar SUVmax and BM fibrosis grade |
| Ayubcha, 2020 [34] | Retrospective, cross-sectional, case-control | Consecutive untreated PV pt., excl. in case of other BM activity-changing diseases | ^18^F-FDG PET/CT | 6 PV, 6 age- and sex- matched controls | Significantly higher skeletal SUVmean in PV pt. compared to controls |
| Agool, 2006 [35] | Probably prospective?, cross-sectional, case-control | Consecutive pt. with hematological disease | ^18^F-FLT PET | 3 MF, 14 controls with solid malignancies without BM involvement | Axial ^18^F-FLT uptake in MF pt. significantly lower compared to controls, splenic uptake significantly higher |
| Vercellino, 2017 [36] | Prospective, cross-sectional | Consecutive pt. | ^18^F-FLT PET/CT + ^99m^Tc-nanocolloid and ^111^In-Cl3 scintigraphy | 15 MF | 3 uptake patterns:  A (20%): normal axial/spleen uptake with only slight peripheral BM expansion. B (60%): normal/slightly decreased axial uptake with high splenic/peripheral BM uptake. C (20%): low axial uptake with high splenic and peripheral BM uptake.  Sign. correlation between ^18^F-FLT SUVmax and BM fibrosis |
| Ojeda-Uribe, 2016 [37] | Retrospective, cross-sectional | Unclear | ^99m^Tc-nanocolloid and ^111^In-Cl3 SPECT/CT | 6 MF | Low axial uptake of both tracers in all pt. with high peripheral uptake in 5/6  Splenic ^111^In-Cl3 uptake correlated to peripheral uptake in pt. with advanced fibrosis  ^99m^Tc-colloid uptake generally lower than ^111^In-Cl3 uptake |
| Sayle, 1982 [38] | Probably retrospective?, cross-sectional / longitudinal | Unclear | ^99m^Tc-sulfur colloid, ^111^In-Cl3 and ^59^Fe scintigraphy | 11 MF (11 ^111^In-Cl3 and ^59^Fe, 1 ^99m^Tc-sulfur colloid) | Lower axial ^111^In-Cl3 uptake in severe disease (no. unclear)  High ^111^In-Cl3 uptake in 9/11 pt.  Similar ^99m^Tc-colloid distribution in 1 pt.  ‘Excellent correlation’ between clinical response and images during treatment in 6 pt (not quantified)  ‘Good correlation’ between ^111^In-Cl3 uptake and BM cellularity (not quantified)  No correlation between scan grade and effective erythrocyte index |
| Fortynova, 1981 [39] | Probably retrospective?, cross-sectional | Unclear | ^99m^Tc-sulfur colloid and ^111^In-Cl3 scintigraphy | 8 MF (9 scans: 3/9 ^111^In-Cl3 and 6/9 ^99m^Tc-sulfur colloid) | 7/8 pt. with decreased BM cellularity had low/absent axial tracer uptake; 1 pt. in early phase disease had normal ^111^In-Cl3 uptake |
| Aburano, 1992 [40] | Unclear | Unclear | ^99m^Tc-leukocyte and ^111^In-Cl3 scintigraphy | 5 MF | Axial uptake of both tracers low in 3 pt. with hypocellular BM and high in 1 pt. with hypercellular BM  Axial ^111^In-Cl3 uptake high but ^99m^Tc-leukocyte low in 1 pt. with granulocytic hypoplasia and erytroid hyperplasia |
| Baglin, 1991 [41] | Timeline unclear,  cross-sectional, case-control | Unclear | ^99m^Tc-microcolloid and ^99m^Tc-MDP scintigraphy (skeleton), thermography (sternum, vertebrae, tibiae) | 6 MF (5 ^99m^Tc-MDP, 3 ^99m^Tc-microcolloid), unknown no. of controls | Increased peripheral ^99m^Tc-microcolloid uptake  Increased ^99m^Tc-MDP uptake in MF, both <1 minute and on delayed images  Temperature difference between skin overlying bone and adjacent skin in MF (not in controls), results compared to histopathology |
| Schreiner, 1974 [42] | Timeline unclear, cross-sectional | Unclear | ^99m^Tc-sulfur colloid scintigraphy | 3 PV, 2 MF | Increased axial, peripheral and splenic uptake in 2 PV pt, normal uptake in one treated PV pt.  Low axial uptake with increased peripheral and splenic uptake in MF pt. |
| Arrago, 1985 [43] | Timeline unclear,  cross-sectional / longitudinal | Unclear | ^99m^Tc-colloid, ^111^In-Cl3 and ^59^Fe scintigraphy | 55 MF, 15 PV | Splenic ^111^In-Cl3 uptake high in MF pt. (sign. correlation with fibrosis grade), low-/normal in PV pt.  Skeletal ^111^In-Cl3 uptake comparable in MF and PV pt, ^99m^Tc-colloid uptake non-sign. lower in MF  Sign. correlation between uptake of both tracers and cellularity; inverse correlation to fibrosis grade  Decrease in axial uptake in 2 untreated pt.; increase in 2 pt on hydroxyurea  Sign. correlation between sacral ^59^Fe and ^111^In-Cl3 uptake |
| Rain, 1996[44] | Probably retrospective?, cross-sectional | Unclear | ^99m^Tc-colloid and ^111^In-Cl3 scintigraphy | 34 PV (incl. 19 ‘spent phase’), 20 MF | General pattern: increased axial and peripheral uptake of both tracers in PV, normal uptake in PV in complete remission, often low axial ^99m^Tc-colloid uptake followed by low ^111^In-Cl3 uptake in advanced disease (spent phase/MF) |
| Rain, 1993 [45] | Timeline unclear, cross-sectional | Unclear | ^99m^Tc-colloid and ^111^In-Cl3 scintigraphy | 50 MF, 17 ‘spent phase’ PV | ^99m^Tc-colloid uptake generally lower than ^111^In-Cl3 uptake  Axial ^99m^Tc-colloid uptake low in 11/17 PV and 9/10 MF pt.  Axial ^111^In-Cl3 uptake low in 0/17 PV and 2/10 MF.  Peripheral BM extension both in PV and MF, splenic ^111^In-Cl3 uptake weak in PV, high in MF |
| Engstedt, 1958 [46] | Timeline unclear, cross-sectional | Unclear | ^198^Au-colloid scintigraphy | 19 PV, 4 controls with solid malignancy without BM involvement | High hepatic uptake in patients and controls, splenic uptake often higher in PV pt.  Axial uptake difficult to compare, often peripheral BM extension in PV |
| Rudberg, 1993 [47] | Timeline unclear,  cross-sectional, | Probably consecutive?, scans for work-up of polycythemia | ^99m^Tc-colloid scintigraphy | 19 PV, 18 patients with secondary polycythemia | Peripheral BM extension in all PV pt. (vs 11/18 secondary polycythemia pt) Increased axial uptake in 16/19 PV pt (vs 0/18 in secondary polycythemia pt) |
| Huic, 2002 [48] | Probably prospective?, cross-sectional, case-control | Unclear | ^99m^Tc-AGAb scintigraphy | 6 MF, 50 historical healthy controls | Increased splenic uptake and decreased BM uptake in MF, with peripheral extension in 1/6 |
| Ferrant, 1986 [49] | Probably retrospective?, cross-sectional | Probably consecutive? Pt. with available scans | ^52^Fe scintigraphy | 7 PV, 59 MF | Peripheral BM extension in 29% of PV pt. and 37% of MF pt.  Marrow iron uptake in axial and peripheral BM sign. correlated in MF |
| Vallabhajosula, 1989 [50] | Probably prospective?, cross-sectional, case-control | Unclear | ^99m^Tc-LDL and ^99m^Tc-colloid scintigraphy | 2 PV, 3 MF, 3 healthy controls | High splenic and peripheral BM uptake of both tracers in 3 MF and 1 PV pt. with hypercellular BM  Almost normal tracer uptake in 1 PV pt. with hypercellular axial BM |
| van Dyke, 1971 [51] | Timeline unclear,  cross-sectional, case-control | Unclear | ^18^F scintigraphy with fluorokinetic analysis | 11 MF, 4 PV, 27 controls (with or without osseous or hematological abnormalities) | Faster BM tracer accumulation in MF compared to most controls (except for a few specific diseases), Minimum blood flow as percentage of cardiac output 7.8% in MF pt. versus 3.3% in controls |
| Martiat, 1987 [52] | Timeline unclear,  cross-sectional, case-control | Unclear | ^15^O-CO2 PET (pelvis) | 6 PV, 4 MF, 5 healthy controls | Pelvic blood flow significantly increased in PV and MF pt. compared to controls (26.9 and 35.1 versus 10ml/min/100^3^, respectively)  No sign. correlation between blood flow and BM cellularity for total population (including 18 pt. with other hematological malignancies) |
| Lahtinen, 1983 [53] | Timeline unclear,  cross-sectional, case-control | Unclear | Scintigraphy with ^133^Xe washout method | 22 PV, 22 age-matched controls | Sign. increased fractional flow of hematopoietic marrow in all PV pt BM blood flow increased in untreated PV pt. compared to controls aged >55 years |
| Lahtinen, 1982 [54] | Timeline unclear,  cross-sectional, case-control | Unclear | Scintigraphy with ^133^Xe washout method | 7 MF, 7 age-matched controls | Sign. increased mean bone blood perfusion in MF compared to controls, particularly in ‘non-hematopoietic compartment’ |
| Zhang, 1989 [55] | Timeline unclear,  cross-sectional | Unclear | ^99m^Tc-RBC and ^113^In-heat damaged RBC scintigraphy | 17 PV, 23 MF | Sign. correlation between splenic vascularity and spleen size in PV and MF pt, sign. correlation between splenic cellularity and spleen size in MF pt. (not in PV) |

S 1. Siniluoto TM, Hyvarinen SA, Paivansalo MJ, Alavaikko MJ, Suramo IJ. Abdominal ultrasonography in myelofibrosis. *Acta Radiol* 1992; 33:343-346

S 2. Iurlo A, Cattaneo D, Giunta M, et al. Transient elastography spleen stiffness measurements in primary myelofibrosis patients: A pilot study in a single centre. *British Journal of Haematology* 2015; 170:890-892

S 3. Webb M, Shibolet O, Halpern Z, et al. Assessment of Liver and Spleen Stiffness in Patients With Myelofibrosis Using FibroScan and Shear Wave Elastography. *Ultrasound Q* 2015; 31:166-169

S 4. Chen CF, Robinson DE, Wilson LS, Griffiths KA, Manoharan A, Doust BD. Clinical sound speed measurement in liver and spleen in vivo. *Ultrason Imaging* 1987; 9:221-235

S 5. Manoharan A, Chen CF, Wilson LS, Griffiths KA, Robinson DE. Ultrasonic characterization of splenic tissue in myelofibrosis: further evidence for reversal of fibrosis with chemotherapy. *Eur J Haematol* 1988; 40:149-154

S 6. Wilson LS, Robinson DE, Griffiths KA, Manoharan A, Doust BD. Evaluation of ultrasonic attenuation in diffuse diseases of spleen and liver. *Ultrason Imaging* 1987; 9:236-247

S 7. Farmer S, Shanbhogue VV, Hansen S, et al. Bone mineral density and microarchitecture in patients with essential thrombocythemia and polycythemia vera. *Osteoporosis international : a journal established as result of cooperation between the European Foundation for Osteoporosis and the National Osteoporosis Foundation of the USA* 2017; 28:677-685

S 8. Farmer S, Vestergaard H, Hansen S, et al. Bone geometry, bone mineral density, and micro-architecture in patients with myelofibrosis: a cross-sectional study using DXA, HR-pQCT, and bone turnover markers. *Int J Hematol* 2015; 102:67-75

S 9. Diamond T, Smith A, Schnier R, Manoharan A. Syndrome of myelofibrosis and osteosclerosis: a series of case reports and review of the literature. *Bone* 2002; 30:498-501

S 10. Mellibovsky L, Marinoso ML, Cervantes F, et al. Relationship among densitometry, bone histomorphometry, and histologic stage in idiopathic myelofibrosis. *Bone* 2004; 34:330-335

S 11. Pettigrew JD, Ward HP. Correlation of radiologic, histologic, and clinical findings in agnogenic myeloid metaplasia. *Radiology* 1969; 93:541-548

S 12. Takacsi-Nagy L, Graf F. Definition, clinical features and diagnosis of myelobibrosis. *Clinics in haematology* 1975; 4:291-308

S 13. Pawelski S, Kotakowski L, Pawlikowski J, Mietkowska I, Sielczak M. Radiological, haematological and histological analysis of myelosclerosis. *Folia haematologica (Leipzig, Germany : 1928)* 1973; 99:1-9

S 14. Hickling RA. The natural history of chronic non-leukaemic myelosis. *The Quarterly journal of medicine* 1968; 37:267-279

S 15. Chelloul N, Briere J, Laval-Jeantet M, Najean Y, Vorhauer W, Jacquillat C. Prognosis of myeloid metaplasia with myelofibrosis. *Biomedicine / [publiee pour l'AAICIG]* 1976; 24:272-280

S 16. Hodgson JR, Good CA, Hall BE. The roentgenographic aspects of polycythemia vera. *Proceedings of the staff meetings Mayo Clinic* 1946; 21:152-157

S 17. Amano Y, Wakabayashi H, Kumazaki T. MR signal changes in bone marrow of mandible in hematologic disorders. *J Comput Assist Tomogr* 1995; 19:552-554

S 18. Amano Y, Onda M, Amano M, Kumazaki T. Magnetic resonance imaging of myelofibrosis. STIR and gadolinium-enhanced MR images. *Clin Imaging* 1997; 21:264-268

S 19. Sale GE, Deeg HJ, Porter BA. Regression of myelofibrosis and osteosclerosis following hematopoietic cell transplantation assessed by magnetic resonance imaging and histologic grading. *Biol Blood Marrow Transplant* 2006; 12:1285-1294

S 20. Alpdogan O, Budak-Alpdogan T, Bayik M, Akoglu T, Kodalli N, Gurmen N. Magnetic resonance imaging in myelofibrosis. *Blood* 1998; 92:2995-2997

S 21. Kaplan KR, Mitchell DG, Steiner RM, et al. Polycythemia vera and myelofibrosis: correlation of MR imaging, clinical, and laboratory findings. *Radiology* 1992; 183:329-334

S 22. Guckel F, Brix G, Semmler W, et al. Systemic bone marrow disorders: characterization with proton chemical shift imaging. *J Comput Assist Tomogr* 1990; 14:633-642

S 23. Rozman M, Mercader JM, Aguilar JL, Montserrat E, Rozman C. Estimation of bone marrow cellularity by means of vertebral magnetic resonance. *Haematologica* 1997; 82:166-170

S 24. Rozman C, Cervantes F, Rozman M, Mercader JM, Montserrat E. Magnetic resonance imaging in myelofibrosis and essential thrombocythaemia: contribution to differential diagnosis. *Br J Haematol* 1999; 104:574-580

S 25. Luker GD, Nguyen HM, Hoff BA, et al. A Pilot Study of Quantitative MRI Parametric Response Mapping of Bone Marrow Fat for Treatment Assessment in Myelofibrosis. *Tomography (Ann Arbor, Mich)* 2016; 2:67-78

S 26. Jensen KE, Grube T, Thomsen C, et al. Prolonged bone marrow T1-relaxation in patients with polycythemia vera. *Magn Reson Imaging* 1988; 6:291-292

S 27. Jensen KE, Jensen M, Grundtvig P, Thomsen C, Karle H, Henriksen O. Localized in vivo proton spectroscopy of the bone marrow in patients with leukemia. *Magn Reson Imaging* 1990; 8:779-789

S 28. Courcoutsakis N, Spanoudaki A, Maris TG, et al. Perfusion parameters analysis of the vertebral bone marrow in patients with Ph(1)(-) chronic myeloproliferative neoplasms (Ph(neg) MPN): a dynamic contrast-enhanced MRI (DCE-MRI) study. *J Magn Reson Imaging* 2012; 35:696-702

S 29. Katsuya T, Inoue T, Ishizaka H, Aoki J, Endo K. Dynamic contrast-enhanced MR imaging of the water fraction of normal bone marrow and diffuse bone marrow disease. *Radiat Med* 2000; 18:291-297

S 30. Tsujikawa T, Tasaki T, Hosono N, et al. (18)F-FLT PET/MRI for bone marrow failure syndrome-initial experience. *EJNMMI Res* 2019; 9:16

S 31. Smith SR, Martin PA, Davies JM, Edwards RH. Characterization of the spleen by in vivo image guided 31P magnetic resonance spectroscopy. *NMR Biomed* 1989; 2:172-178

S 32. Derlin T, Alchalby H, Bannas P, et al. Assessment of bone marrow inflammation in patients with myelofibrosis: an 18F-fluorodeoxyglucose PET/CT study. *Eur J Nucl Med Mol Imaging* 2015; 42:696-705

S 33. Derlin T, Alchalby H, Bannas P, et al. Serial 18F-FDG PET for Monitoring Treatment Response After Allogeneic Stem Cell Transplantation for Myelofibrosis. *Journal of nuclear medicine : official publication, Society of Nuclear Medicine* 2016; 57:1556-1559

S 34. Ayubcha C, Hosoya H, Mehdizadeh Seraj S, Zirakchian Zadeh M, M SET, Alavi A. A Pilot Study of FDG-PET/CT in Polycythemia Vera Using Global Analysis Techniques. *Asia Ocean J Nucl Med Biol* 2020; 8:64-68

S 35. Agool A, Schot BW, Jager PL, Vellenga E. 18F-FLT PET in hematologic disorders: a novel technique to analyze the bone marrow compartment. *J Nucl Med* 2006; 47:1592-1598

S 36. Vercellino L, Ouvrier MJ, Barre E, et al. Assessing Bone Marrow Activity in Patients with Myelofibrosis: Results of a Pilot Study of (18)F-FLT PET. *Journal of nuclear medicine : official publication, Society of Nuclear Medicine* 2017; 58:1603-1608

S 37. Ojeda-Uribe M, Morel O, Ungureanu C, Desterke C, Le Bousse-Kerdiles MC, Boulahdour H. Assessment of sites of marrow and extramedullary hematopoiesis by hybrid imaging in primary myelofibrosis patients. *Cancer medicine* 2016; 5:2378-2384

S 38. Sayle BA, Helmer RE, 3rd, Birdsong BA, Balachandran S, Gardner FH. Bone-marrow imaging with indium-111 chloride in aplastic anemia and myelofibrosis: concise communication. *Journal of nuclear medicine : official publication, Society of Nuclear Medicine* 1982; 23:121-125

S 39. Fortynova J, Bakos K, Pradacova J. Bone marrow scintigraphy in hemopoietic depletion states. *Czechoslovak medicine* 1981; 4:137-146

S 40. Aburano T, Yokoyama K, Shuke N, et al. Tc-99m HMPAO-labeled leukocytes for hematopoietic marrow imaging. Comparison with In-111 chloride. *Clin Nucl Med* 1992; 17:938-944

S 41. Baglin TP, Crocker J, Timmins A, Chandler S, Boughton BJ. Bone marrow hypervascularity in patients with myelofibrosis identified by infra-red thermography. *Clin Lab Haematol* 1991; 13:341-348

S 42. Schreiner DP. Reticuloendothelial scans in disorders involving the bone marrow. *Journal of nuclear medicine : official publication, Society of Nuclear Medicine* 1974; 15:1158-1162

S 43. Arrago JP, Rain JD, Vigneron N, et al. Diagnostic value of bone marrow imaging with 111indium-transferrin and 99m technetium-colloids in myelofibrosis. *Am J Hematol* 1985; 18:275-282

S 44. Rain JD, Najean Y, Billotey C. Bone marrow scintigraphy as a useful method for estimating the physiological status of bone marrow and spleen in polycythaemia vera. *Leukemia & lymphoma* 1996; 22 Suppl 1:105-110

S 45. Rain JD, Najean Y. Bone marrow scintigraphy in myelofibrosis. *Nouvelle revue francaise d'hematologie* 1993; 35:101-102

S 46. Engstedt L, Franzen S, Jonsson L, Larsson LG. In vivo localization of colloidal Au198 intravenously injected in polycythemia vera; a preliminary report. *Acta radiol* 1958; 49:66-71

S 47. Rudberg U, Skarberg KO. RES scintigraphy in polycythemia vera and secondary or relative polycythemia. *Acta Radiol* 1993; 34:183-186

S 48. Huic D, Ivancevic V, Aurer I, et al. Bone marrow immunoscintigraphy in haematological patients with pancytopenia: preliminary results. *Nuclear medicine communications* 2002; 23:757-763

S 49. Ferrant A, Rodhain J, Leners N, et al. Quantitative assessment of erythropoiesis in bone marrow expansion areas using 52Fe. *Br J Haematol* 1986; 62:247-255

S 50. Vallabhajosula S, Gilbert HS, Goldsmith SJ, Paidi M, Hanna MM, Ginsberg HN. Low-density lipoprotein (LDL) distribution shown by 99mtechnetium-LDL imaging in patients with myeloproliferative diseases. *Ann Intern Med* 1989; 110:208-213

S 51. Van Dyke D, Parker H, Anger HO, et al. Markedly increased bone blood flow in myelofibrosis. *Journal of nuclear medicine : official publication, Society of Nuclear Medicine* 1971; 12:506-512

S 52. Martiat P, Ferrant A, Cogneau M, et al. Assessment of bone marrow blood flow using positron emission tomography: no relationship with bone marrow cellularity. *Br J Haematol* 1987; 66:307-310

S 53. Lahtinen R, Lahtinen T, Hyodynmaa S. Increased bone marrow blood flow in polycythemia vera. *European journal of nuclear medicine* 1983; 8:19-22

S 54. Lahtinen R, Lahtinen T, Romppanen T. Bone and bone-marrow blood flow in chronic granulocytic leukemia and primary myelofibrosis. *Journal of nuclear medicine : official publication, Society of Nuclear Medicine* 1982; 23:218-224

S 55. Zhang B, Lewis SM. The splenomegaly of myeloproliferative and lymphoproliferative disorders: splenic cellularity and vascularity. *Eur J Haematol* 1989; 43:63-66
